# Moderate Perinatal Choline Deficiency Elicits Altered Physiology and Metabolomic Profiles in the Piglet

**DOI:** 10.1371/journal.pone.0133500

**Published:** 2015-07-21

**Authors:** Caitlyn M. Getty, Ryan N. Dilger

**Affiliations:** 1 Division of Nutritional Sciences, University of Illinois, Urbana, Illinois, United States of America; 2 Department of Animal Sciences, University of Illinois, Urbana, Illinois, United States of America; 3 College of Veterinary Medicine, University of Illinois, Urbana, Illinois, United States of America; 4 Neuroscience Program, University of Illinois, Urbana, IL, United States of America; INRA, FRANCE

## Abstract

Few studies have evaluated the impact of dietary choline on the health and well-being of swine, and those pivotal papers were aimed at determining dietary requirements for sows and growing pigs. This is of importance as the piglet is becoming a widely accepted model for human infant nutrition, but little is known about the impacts of perinatal choline status on overall health and metabolism of the growing piglet. In the present study, sows were provided either a choline deficient (CD, 625 mg choline/kg dry matter) or choline sufficient (CS, 1306 mg choline/kg dry matter) diet for the last 65 d of gestation (prenatal intervention). Piglets were weaned from the sow 48 h after farrowing and provided either a CD (477 mg choline/kg dry matter) or CS (1528 mg choline/kg dry matter) milk replacer (postnatal intervention) for 29 ± 2 d, resulting in a factorial arrangement of 4 treatment (prenatal/postnatal) groups: CS/CS, CS/CD, CD/CS, and CD/CD. Piglet growth was normal for artificially-reared piglets, and was not impacted by perinatal choline status. Piglets receiving the postnatal CD treatment had lower (*P* < 0.01) plasma choline and choline-containing phospholipid concentrations and higher (*P* < 0.05) liver enzyme (alkaline phosphatase and gamma-glutamyl transferase) values compared with piglets receiving the postnatal CS treatment. Hepatic lipid content of piglets receiving the postnatal CD treatment was higher (*P* < 0.01) compared with piglets receiving the postnatal CS treatment. Additionally, postnatally CD piglets had lower (*P* = 0.01) plasma cholesterol than postnatally CS piglets. Brain development was also impacted by perinatal choline status, with brains of piglets exposed to prenatal CD being smaller (*P* = 0.01) than those of prenatally CS piglets. These findings support the hypothesis that the piglet is a sensitive model for choline deficiency during the perinatal period. In the present study, piglets exhibited similarities in health markers and metabolomic profiles to rodents and humans when exposed to moderate choline deficiency.

## Introduction

Choline is an essential dietary nutrient in humans and animals; however, 90% of adults consume choline at or below the adequate intake level [1–3]. Of the nearly 900 women participating in a maternal nutrient intake study (Project Viva), 85% were consuming choline below the adequate intake (AI, 450 mg/d) [2] during the first and second trimesters [4]. Moreover, evidence suggests choline consumption by pregnant women may even be less than 400 mg/d [5]. To compound this problem, recent studies [6, 7] have shown that recommended intake levels may be set too low for pregnant women.

Though knowledge regarding choline and neonatal development in humans is lacking, it has been well-documented that sufficient choline intake remains important throughout life. Choline is required for cell membrane synthesis, export of very low density lipoprotein (VLDL) from the liver, myelin synthesis, neurotransmitter synthesis, and one-carbon metabolism. While the body can synthesize phosphatidylcholine *de novo* via the phosphatidylethanolamine *N*-methyltransferase (PEMT) pathway, this metabolic route is insufficient to meet physiological requirements [2]. Choline deficiency during adulthood results in liver dysfunction, non-alcoholic fatty liver disease, and liver cell death due to the inability of the liver to package and excrete VLDL [8, 9]. Metabolomic analyses have recently provided evidence that adults subjected to choline deficiency exhibit altered fatty acid metabolism, as well as liver and kidney function [10].

To date, most studies exploring the impact of perinatal choline deficiency on metabolism and neurodevelopment have been conducted in rodents. However, there are several differences between rodents and humans which make translation of nutritional effects difficult. Utilizing an animal model that possesses anatomy and physiology more similar to humans may provide novel insights into perinatal choline status. To this end, the pig may be an ideal animal model to study the effects of perinatal choline deficiency. Humans and pigs share striking similarities in anatomy, physiology, metabolism, nutrient requirements, and neurodevelopment [11–14]. As a precocial species, pigs are mobile and can be easily weaned onto an artificial milk replacer immediately following birth [15–17], which allows precise control over perinatal choline status in a way that has not yet been accomplished in rodents, and cannot ethically be conducted in human infants.

Considering use of the pig has distinct advantages in the study of metabolism, our objective was to characterize how moderate prenatal and postnatal choline deficiency influenced piglet growth, overall health status, and metabolic profiles. Based on previous research involving perinatal choline deficiency, we hypothesized that piglets exposed to moderate choline deficiency would exhibit abnormal metabolic patterns compared with piglets exposed to sufficient perinatal choline supply.

## Materials and Methods

### Ethics Statement

All animal care and experimental procedures were in accordance with the Guide for the Care and Use of Laboratory Animals and approved by the Institutional Animal Care and Use Committee of the University of Illinois (Protocol 10125).

### Animals and Housing

Eight multiparous Yorkshire sows (University of Illinois Imported Swine Research Laboratory herd), bred to either Duroc (n = 7) or Yorkshire (n = 1) boars, were housed in standard gestation and farrowing crates for the duration of this experiment. Beginning on d 50 of the 114 d gestation period, sows were assigned to one of two experimental diets (prenatal intervention) containing either a sufficient (CS) or deficient (CD) dietary concentration of choline. This feeding period was chosen in order to minimize risk of fetal loss due to choline deficiency [18, 19], is the gestational period most relevant to brain development [14], and mimics numerous rodent studies evaluating choline status and hippocampal development [20–22].

Two replicates of this experiment, using 2 CS and 2 CD sows each, were performed using consecutive farrowing groups. Sows were fed once each day (0700 h) to maintain body condition and were allowed *ad libitum* access to water. Experimental gestation diets were provided until 48 h after farrowing. Prior to provision of experimental diets (d 50 of gestation) and again within 48 h after giving farrowing, blood was collected from sows via jugular venipuncture into heparinized tubes between 1300 and 1500 when post-prandial plasma choline concentrations had stabilized [23]. Blood was centrifuged at 1,300 × g for 10 min at 4°C and plasma stored at -80°C until analyzed.

A total of 32 piglets (n = 8 per treatment group, with 4 female and 4 intact male piglets per group; 16 piglets per replicate) were assigned to one of two custom milk replacer formulations (postnatal intervention): CD or CS. Between the prenatal (i.e., sow gestation diets) and postnatal (i.e., piglet milk replacers) treatments, this study employed a total of 4 treatment groups: CS/CS, prenatal and postnatal choline sufficient; CS/CD, prenatal choline sufficient and postnatal choline deficient; CD/CS, prenatal choline deficient and postnatal choline sufficient; and CD/CD, prenatal and postnatal choline deficient. Piglets were assigned to treatment groups by evenly distributing genetics, sex, and weight. Starting piglet weights for each treatment group were as follows: CS/CS, 1.79 ± 0.06 kg; CS/CD, 1.77 ± 0.06 kg; CD/CS, 1.72 ± 0.06 kg; CD/CD, 1.75 ± 0.06 kg.

Piglets were moved to the artificial rearing system at 48 h of age, which allowed all piglets to receive colostrum, and immediately provided postnatal experimental treatments.The artificial rearing system was similar to what was previously described [16, 17], with the following differences: stainless steel cages measured (1.03 m deep x 0.77 m wide x 0.81 m high) and had multiple 2.54 cm diameter holes drilled in the sides for ventilation with clear, Plexiglas doors, and a towel and a soft rubber toy were provided for enrichment. Climate was maintained between 23–31° C using a combination of heat lamps and electric heat mats, and a 12 h-light/12 h-dark cycle was maintained with light from 0600 to 1800. Piglets were weighed individually each morning. If piglets developed diarrhea, they were placed on an electrolyte solution and provided supplemental water, and if the diarrhea did not resolve within 48 h, piglets received a single dose of ceftiofur (5.0 mg ceftiofur equivalent/kg of body weight i.m [Excede, Zoetis, Florham Park, NJ]). If fluid loss continued after treatment, piglets then received a single dose of sulfamethoxazole and trimethoprim oral suspension (50 mg/8 mg per mL, Hi-Tech Pharmacal, Amityville, NY) for 3 consecutive days. Because of the unseasonably warm weather when this study was conducted (31.8° C [2012] vs. 28.7° C [historical average]), sows were allowed to farrow without induction, which resulted in final piglets ages between 27–30 d.

### Dietary Treatments

Corn and soy protein isolate-based sow diets were formulated to meet requirements for all nutrients [24], except choline (Table 1). From d 50 of gestation through 48 h after farrowing, sows received either a choline sufficient diet (analyzed as 1,306 mg of choline/kg of DM) or a choline deficient diet (analyzed as 625 mg of choline/kg of DM). On d 94 of gestation, a prophylactic antibiotic (BMD 60, Alpharma, Bridgewater, NJ) was added to sow diets according to manufacturer specifications to prevent Clostridium perfringens diarrhea in piglets.

**Table 1:** Composition of gestation diet (choline-deficient)^1^.

Ingredient	g/kg
Ground corn	785.0
Molassed dried sugar beet pulp	70.0
Soy protein isolate^2^	60.0
Cornstarch	42.7
Dicalcium phosphate	20.0
Corn oil	10.0
Limestone	7.5
Vitamin and mineral premix^3^	3.0
DL-Met	1.5
L-Trp	0.3

^1^Choline sufficient diet was produced by replacing 1.8 g of cornstarch per kg of final diet with choline chloride (containing 60% choline). Choline deficient and sufficient gestation diets were analyzed to contain 625 and 1,306 mg of choline per kg diet, respectively.

^2^Ardex F, Archer Daniels Midland, Decatur, IL.

^3^Provided per kg of complete diet: Ca, 0.6 mg (CaCO_3_); P, 298.8 mg; Mg, 1.8 mg; K, 2.4 mg; Na, 0.3 mg; S, 172.2 mg; Zn, 125.1 mg; Fe, 129.1 mg (FeSO_4_); Mn, 60.3 mg (MnSO_4_); Cu, 10.2 mg (CuCl_2_, CuSO_4_); I, 1.3 mg; Se, 0.3 mg (Na_2_SeO_3_); Cl, 2.1 mg; Vit A, 11.1 kIU; Vit D, 2.2 kIU; Vit E, 66.1 kIU; Vit K, 1.4 mg (menadione dimethylpyrimidinol bisulfite); thiamin, 0.2 mg (thiamine mononitrate); riboflavin, 6.6 mg; niacin, 44.1 mg; pantothenic acid, 23.5 mg (D-calcium pantothenate); pyridoxine, 0.2 mg (pyridoxine-HCl); biotin, 0.4 mg; folacin, 1.6 mg; Vit B12, 0.03 mg.

Powdered soy-based milk replacer formulas (Test Diet, St. Louis, MO) were formulated to meet requirements of all nutrients [24], except choline, for the young pig (Table 2). The primary protein source was a human food-grade, dispersible soy protein isolate (Ardex F, Archer Daniels Midland, Decatur, IL). Upon arrival to the animal care facility at approximately postnatal day (PD) 2, piglets received either a choline sufficient formula (analyzed as 1,528 mg of choline/kg of DM) or a choline deficient formula (analyzed as 477 mg of choline/kg of DM) for the duration of the feeding study. It is important to note that the CS milk replacer provided a similar concentration of choline (306 mg/L) as sow’s milk (364 mg/L) [25]. Choline concentration of the sow’s milk in this study was not measured. Milk replacer formula was reconstituted fresh at least 4 times daily to a final concentration of 200 g of milk replacer powder per L using tap water. The liquid milk replacer was then supplied at 285 mL/kg of body weight for study d 0–10, 300 mL/kg body weight for study d 11–16, and 325 mL/kg body weight for study d 17–30. Feeding rates and intervals (every 2–4 h, beginning at 1000 and ending at 0200 the following morning) were increased based on growth performance of the piglets being fed the choline sufficient milk replacer.

**Table 2:** Composition of milk replacer formulation (choline deficient)^1^.

Ingredient	g/kg
Lactose	425.6
Dried fat 7/60^2^	244.8
Soy protein isolate^3^	202.5
L-Lys HCl	30.0
Dicalcium phosphate	20.0
Calcium carbonate	19.8
Potassium citrate, tribasic monohydrate	18.8
Vitamin and mineral premix^4^	11.8
Salt	11.3
Potassium sorbate	10.0
L-Cys	2.0
DL-Met	1.5
Powdered cellulose^5^	0.9
Palatant^6^	0.8
Calcium chloride	0.2

^1^Choline sufficient milk replacer was produced by replacing 2.2 g lactose and 0.22g calcium chloride with choline chloride (containing 70% choline). Choline deficient and sufficient milk replacer formulations were analyzed to contain 477 and 1,528 mg of choline per kg diet, respectively.

^2^Ho-Milc 7–60, Merrick’s, Inc., Middleton, WI.

^3^Ardex F, Archer Daniels Midland, Decatur, IL.

^4^Provided per kg of complete diet: Cu, 23.5 mg (CuSO_4_); Zn, 257.2 mg (ZnSO_4_); Se, 1.0 mg (Na_2_SeO_3_); Mg, 2.88 g (MgCO_3_); NaPO_4_, 5.2 g; menadione, 5.1 mg; Vit C, 51.0 mg; Vit A, 47.0 mg; Vit D, 918.6 mg; Vit E, 673.6 mg; thiamin, 29.6 mg (10%); riboflavin, 102.1 mg; niacin, 62.3 mg (98%); biotin, 316.4 mg; Ca-pantothenate, 188.8 mg; pyridoxine, 306.2 mg; folate, 47.0 mg; Vit B12, 170.4 mg.

^5^Solka Floc, International Fiber Corporation, North Tonawanda, NY.

^6^Luctarom Milky Vanilla, Lucta USA, Inc., Northbrook, IL.

### Blood and Tissue Collection

At 4 weeks of age, piglets were anesthetized using a telazol:ketamine:xylazine solution (50.0 mg of tiletamine plus 50.0 mg of zolazepam reconstituted with 2.50 mL ketamine [100 g/L] and 2.50 mL xylazine [100 g/L]; Zoetis, Florham Park, NJ). The anesthetic combination was administered i.m. at 0.03 mL/kg body weight. After verifying anesthetic induction, piglets were euthanized via intracardiac administration of sodium pentobarbital (86.0 mg/kg of body weight; Fatal Plus; Vortech Pharmaceuticals, Dearborn, MI). Immediately following determination of death, heads were removed, and trunk blood was collected for plasma (both lithium heparinized and EDTA blood were collected) and serum, prior to removing, weighing, and dissecting whole brains and livers from piglets. All tissue samples were immediately snap-frozen in liquid nitrogen or preserved in RNA*later* (Ambion, Grand Island, NY), and stored at -80° C until processing.

### Laboratory Analyses

#### Dietary Treatments

Gestation diet and milk replacer samples were analyzed for dry matter (AOAC, 934.01), organic matter and ash (AOAC, 942.05), amino acids (AOAC, 982.30), crude fat (by ether extract, AOAC, 920.39), and crude protein (by combustion analysis of total nitrogen; Leco Corp., St. Joseph, MI; assumed a correction factor of 6.25, AOAC, 990.03) by the University of Missouri Agricultural Experiment Station Chemical Laboratories. Diet samples were also analyzed for gross energy by adiabatic bomb calorimetry (Parr Instruments, Moline, IL). Additionally, all experimental diets were analyzed for folic acid (AOAC, 1990) and choline (AOAC, 999.14) by an external laboratory (Eurofins US, Des Moines, IA). All dietary analytical information can be found in Table 3.

**Table 3:** Analyzed composition of sow gestation diets and piglet milk replacers^1^.

	Sow Gestation Diets		Piglet Milk Replacers	
Item	Deficient	Sufficient	Deficient	Sufficient
Dry matter, %	87.33	87.32	96.73	96.87
	————————————g/100 g of dry matter————————————			
Organic matter	95.35	95.57	91.56	91.63
Crude protein	13.51	13.64	24.40	23.96
Crude fat	4.35	3.39	15.26	15.67
Gross energy, kcal/g	4.43	4.37	4.68	4.84
Choline, mg/kg	625	1,306	477	1,528
Folic acid, mg/kg	1.43	1.45	1.21	1.01
Amino acids				
Arg	0.78	0.76	1.31	1.40
Cys	0.21	0.21	0.34	0.50
Iso	0.54	0.53	0.94	1.01
Leu	1.26	1.26	1.56	1.65
Lys	0.63	0.62	4.31	4.16
Met	0.39	0.36	0.29	0.26
Phe	0.65	0.64	1.00	1.06
Thr	0.44	0.44	0.69	0.74
Trp	0.15	0.15	0.33	0.32
Val	0.68	0.65	1.02	1.08

^1^Experimental diets were formulated to meet or exceed requirements for sows and piglets [24].

#### Lipid Analysis

Choline and phospholipid content of heparinized plasma were assayed using a colorimetric procedure (Phospholipid Assay Kit, KA1635; Abnova, Taipei City, Taiwan). The assay was run according to manufacturer instructions, and colorimetric results were determined using a plate reader (Biotek, Winooski, VT). Briefly, choline was cleaved from choline-containing phospholipids by phospholipase D, and the resulting choline was subsequently oxidized to betaine by choline oxidase. Evolved H_2_O_2_ concentrations were then quantified using horseradish peroxidase to produce color at 570 nm. Colorimetric results were plotted against a standard curve generated by subjecting serial dilutions of phosphatidylcholine to the same protocol (detection range, 3–200 μmol/L; intra-assay CV, 2.3%). Liver samples were analyzed for dry matter (AOAC, 934.01) and acid hydrolyzed fat (AOAC 922.06), the intra-assay CV for acid hydrolyzed fat was 9.4%

#### Blood Chemistry and Complete Blood Count

Whole blood (heparinized and EDTA) from euthanized piglets was submitted to the University of Illinois College of Veterinary Medicine Animal Diagnostic Laboratory for blood chemistry analysis, which included: creatinine, blood urea nitrogen (BUN), total protein, albumin, calcium, phosphorus, sodium, potassium, chloride, glucose, alkaline phosphatase (ALP), aspartate aminotransferase (AST), gamma-glutamyltransferase (GGT), total bilirubin, creatine kinase, cholesterol, CO_2_, glutamate dehydrogenase (GLDH), and magnesium. A complete blood count with differential was also performed by trained staff using a combination of automated and manual procedures.

#### Metabolomics

Plasma (derived from EDTA whole blood) was submitted for global, non-targeted metabolomics analyses (Metabolon, Inc., Durham, NC). Briefly, samples were processed using the MicroLab STAR system (Hamilton Company, Reno, NV), which included the addition of recovery standards, protein fraction removal, and removal of organic solvents. Samples were then prepared for either liquid chromatography/mass spectrometry (LC/MS) electrospray ionization tandem mass spectroscopy or gas chromatography/mass spectrometry (GC/MS) procedures. Protocols for LC/MS [26] and GC/MS [27] were previously described with the following GC/MS deviation: the initial oven temperature was 40° C ramped to 300° C in a 16 min period. Compounds were identified by comparison to library entries of purified standards or recurrent unknown entries. Identification of known chemical entities was based on comparison to metabolomic library entries of purified standards. At the time of analysis, there were more than 1,000 commercially available standards with which to compare.

### Statistical Analysis

Data analysis was conducted using the MIXED procedure of SAS (SAS Inst. Inc., Cary, NC). Sow performance data (litter size, birth weight, and piglets lost) were analyzed as a 1-way ANOVA (prenatal choline status) with sow as the experimental unit. Growth data were analyzed as a 3-way repeated measures ANOVA (prenatal choline status, postnatal choline status, and day as the repeated measure), with farrowing group as a blocking factor. All other data were analyzed as a 2-way ANOVA with main effects of prenatal choline status, and postnatal choline status, with farrowing group as a blocking factor. Significance was accepted at *P* < 0.05, with trends denoted at 0.05 < *P* < 0.10. Data are presented as least square means ± SEM. Individual piglet was considered the experimental unit.

## Results

### Sow and Piglet Performance Measures

All sows carried their pregnancy to term, and there were no differences in litter size (CS, 8.3 ± 1.6 piglets; CD, 9.0 ± 1.6 piglets; *P* = 0.75), piglet birth weights (CS, 1.59 ± 0.04 kg; CD, 1.52 ± 0.04 kg; *P* = 0.22), or the number of piglets that were born dead or had been laid on (CS, 0.8 ± 0.7 piglets; CD, 2.5 ± 0.7 piglets; *P* = 0.15) at the time of processing between CS and CD sows. Additionally, plasma choline-containing phospholipid concentrations did not differ (*P* = 0.48) at the time of farrowing with 588 ± 61 μmol/L or 654 ± 61 μmol/L in sows fed sufficient or deficient gestation diets, respectively.

**Fig 1:**
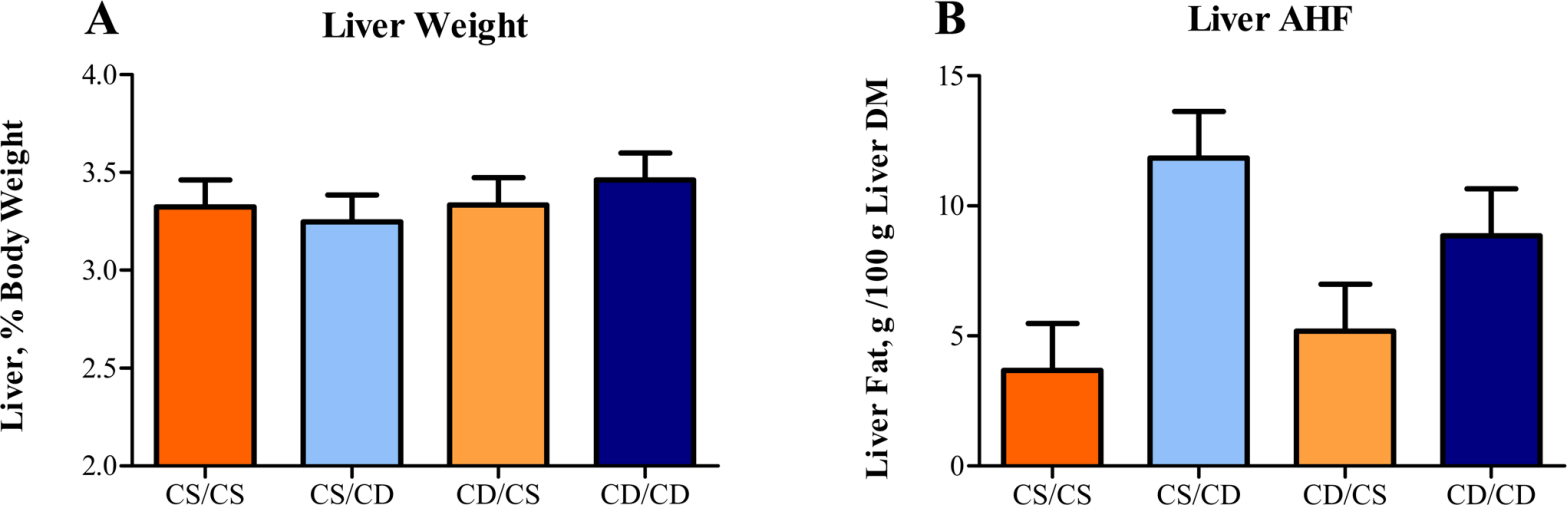
Effects of perinatal choline status on final body weights and brain size of piglets. Values are means of 8 replicate pigs exposed to prenatal and postnatal choline treatments (e.g., CS/CS as the control group). A) Piglet weights at 27 d of age did not differ between treatments. B) Regardless of postnatal choline status, pigs exposed to prenatal CD environment had smaller (*P* = 0.01) brains than those piglets exposed to a prenatal CS environment. Abbreviations: CD, choline deficient; CS, choline sufficient

Body weight gain of piglets was normal for piglets raised in an artificial rearing system [15–17, 28, 29]. There was no difference (*P* = 0.48) in mean starting weight of piglets among the 4 perinatal treatments (Fig 1), and piglets exhibited no differences in rate of body weight gain (*P* = 0.90) or overall body weight at 27 d of age (*P* = 0.35). Interestingly, perinatal choline status impacted brain growth in neonatal piglets (Fig 1). A main effect of prenatal choline status (*P* = 0.01) was observed for relative brain weights of piglets at PD 27–30, where prenatally CS piglets had larger brains than prenatally CD piglets.

### Piglet plasma choline and chemistry profiles

**Fig 2:**
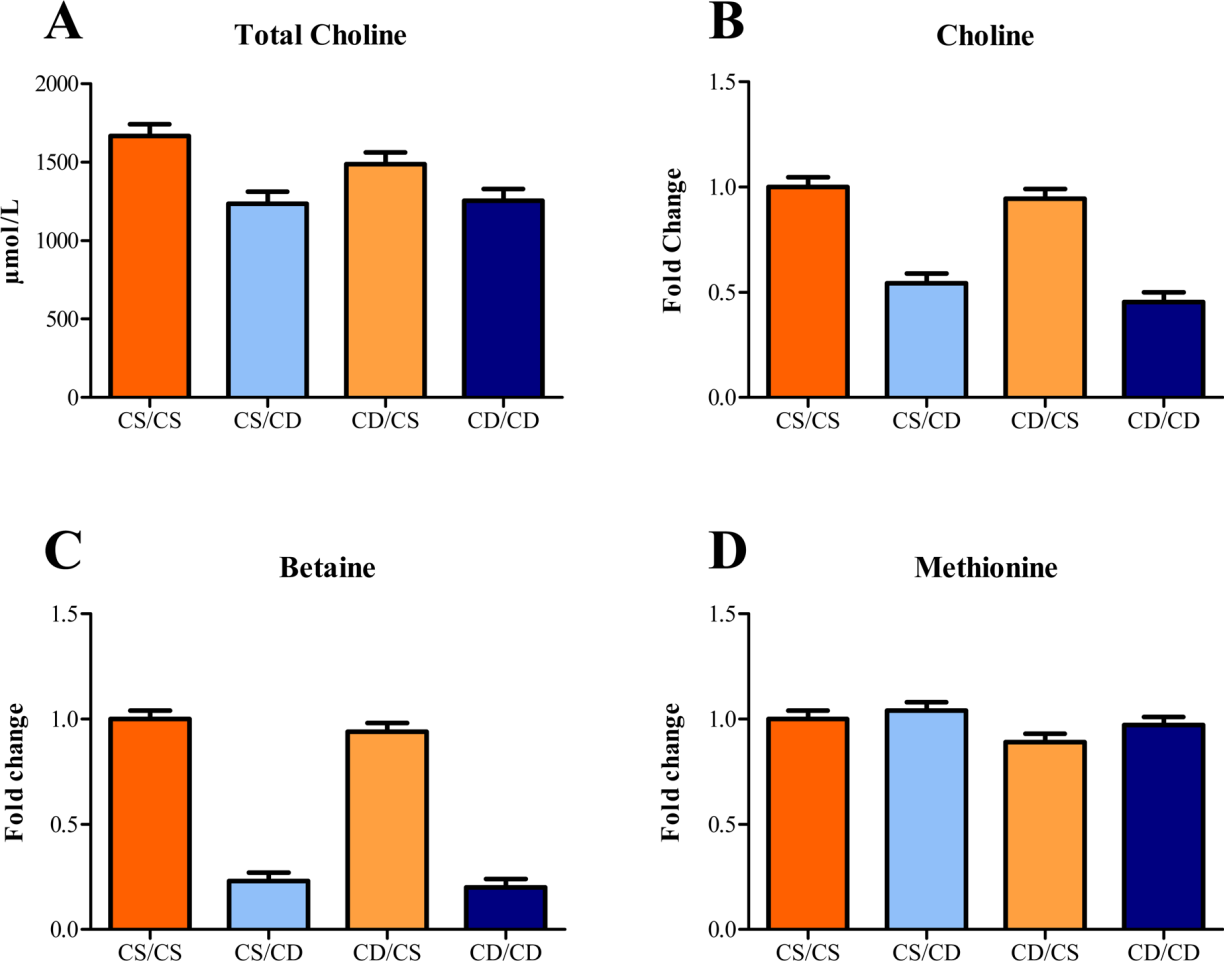
Effects of perinatal choline status on relative concentrations of metabolites related to methyl metabolism in 4-wk-old piglets. Values are means of 8 replicate pigs exposed to prenatal and postnatal choline treatments (e.g., CS/CS as the control group) with blood samples collected at 27–30 d of age. Regardless of prenatal choline status, pigs fed a CD milk replacer exhibited lower (*P* < 0.01) plasma concentrations of: A) total choline + choline-containing phospholipids, B) choline, and C) betaine, compared with pigs fed CS milk replacer. D) Plasma methionine in piglets exposed to prenatal CD environment was lower (*P* = 0.05) compared with piglets exposed to a prenatal CS environment, regardless of postnatal choline status. Abbreviations: CD, choline deficient; CS, choline sufficient

Postnatal choline status significantly impacted plasma free choline + choline-containing phospholipid concentrations in piglets (Fig 2A), where postnatally CD piglets had lower (*P* < 0.01) concentrations than postnatally CS piglets, regardless of prenatal choline status. There was a main effect of postnatal choline status on plasma ALP and GGT, where postnatally CD piglets had higher (*P* = 0.02) values of both enzymes when compared with postnatally CS piglets (Table 4). An interactive effect (*P* = 0.01) was observed for AST, where values for CD/CD and CD/CS piglets were not different, but were higher than for CS/CS piglets, whereas CD/CD piglets were not different than any other group. However, this effect may be due in part to a similar interactive effect observed for hemolysis of blood samples. There was a main effect of postnatal choline status on plasma cholesterol concentrations where postnatally CD piglets had lower (*P* = 0.01) concentrations than postnatally CS piglets. Moreover, there was an interactive effect (*P* = 0.02) of perinatal choline status on creatine kinase where CS/CD and CD/CS piglets tended to have greater concentrations than CS/CS and CD/CD piglets. Additionally, prenatally CS piglets had higher (*P* = 0.02) plasma immunoglobulins than prenatally CD piglets, without an accompanying change in plasma albumin.

**Table 4:** Effects of perinatal choline status on clinical blood chemistry profiles of 4-wk-old piglets^1^.

	Treatment (Prenatal/Postnatal)					*P*-value^2^		
Variable	CS/CS	CS/CD	CD/CS	CD/CD	SEM	Pre	Post	Pre x Post
Total protein, g/L	36	38	35	36	1.3	0.23	0.36	0.64
Albumin, g/L	18	19	20	19	0.6	0.06	0.61	0.06
Immunoglobulins, g/L	19	19	15	17	1.2	**0.02**	0.43	0.63
Glucose, mmol/L	9.10	7.58	7.22	7.51	0.64	0.14	0.34	0.17
ALP, U/L	366.4	485.9	392.8	489.0	42.6	0.73	**0.02**	0.79
AST, U/L	53.9^a^	87.1^b^	88.6^b^	57.6^ab^	11.2	0.82	0.92	**0.01**
GGT, U/L	28.0	31.9	29.1	39.1	2.81	0.15	**0.02**	0.28
GLDH, U/L	0.59	0.64	0.54	0.41	0.15	0.36	0.80	0.56
Cholesterol, mmol/L	2.11	1.80	2.27	1.98	0.11	0.16	**0.01**	0.94
Total bilirubin, μmol/L	4	6	6	7	1	0.35	0.28	0.80
Creatinine, μmol/L	48	48	48	47	2	0.82	0.82	0.82
BUN, mmol/L	3.3	3.4	3.3	2.9	0.2	0.19	0.40	0.19
Creatine kinase, U/L	1527	2799	2700	1482	480	0.88	0.96	**0.02**

^ab^Means within a row and without a common superscript differ (*P* < 0.05).

^1^Values are means of 8 replicate pigs exposed to prenatal and postnatal choline treatments (e.g., CS/CS as the control group) with blood collected from piglets at 27–30 d of age. ALP, alkaline phosphatase; AST, aspartate aminotransferase; BUN, blood urea nitrogen; CD, choline deficient; CS, choline sufficient; GLDH, glutamate dehydrogenase; GGT, gamma-glutamyl transferase.

^2^Pre, main effect of prenatal choline status; Post, main effect of postnatal choline status; Pre x Post, interactive effect of prenatal and postnatal choline statuses.

### Piglet CBC profiles

Perinatal choline deficiency minimally impacted complete blood count (CBC) profiles of piglets (S1 Table). There were no differences in any red blood cell (RBC) parameter (total RBC, hematocrit [Hct], hemoglobin [Hb], or mean corpuscular hemoglobin [MCH]) except for mean corpuscular hemoglobin concentration (MCHC), which exhibited a main effect of prenatal choline status, with prenatally CS piglets having lower (*P* = 0.05) values than prenatally CD piglets. There were no differences in any white blood cell (WBC) parameter (total WBC, total lymphocytes, total monocytes, total eosinophils, or total basophils) except for total neutrophil concentration, which exhibited a main effect of postnatal choline status, with postnatally CS piglets having more (*P* = 0.04) neutrophils than postnatally CD piglets (data not shown). Finally, there were no effects of perinatal choline status on total platelet concentration.

### Hepatic Lipid Content

**Fig 3:**
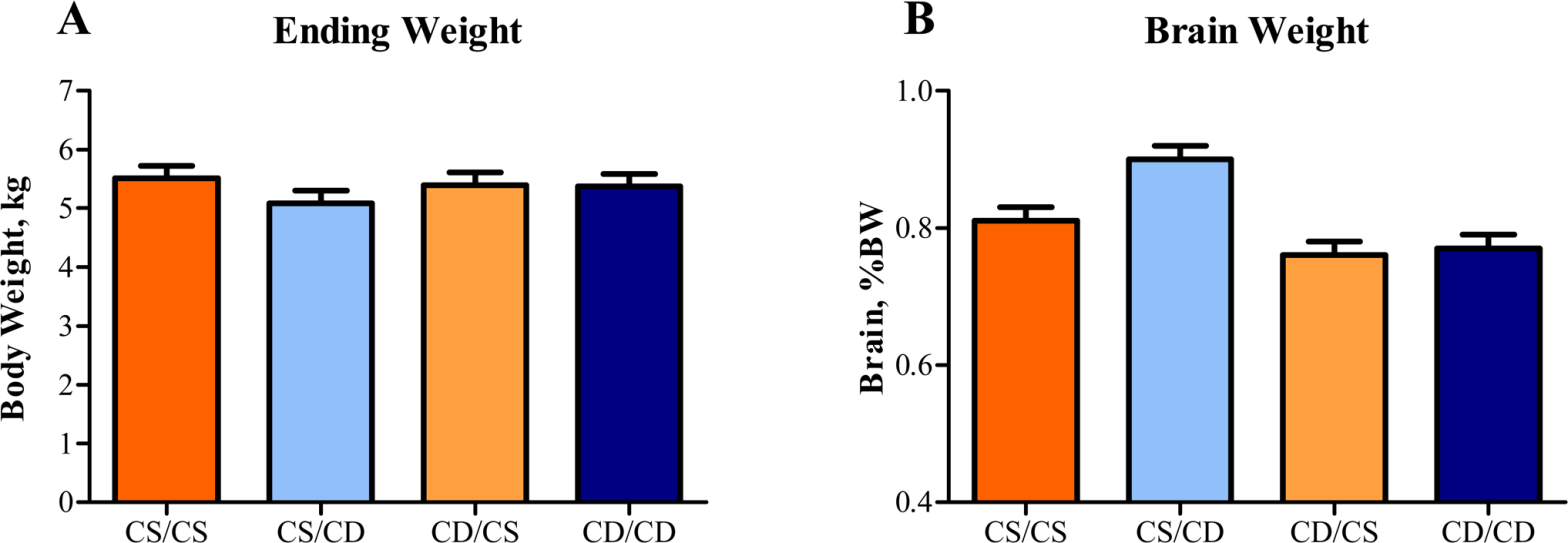
Effects of perinatal choline status on liver weight and lipid composition in 4-wk-old piglets. Values are means of 8 replicate pigs exposed to prenatal and postnatal choline treatments (e.g., CS/CS as the control group) with tissue samples collected at 27–30 d of age. A) Relative liver weight was not different between perinatal choline treatments, B) Piglets fed CD milk replacer exhibited higher (*P* < 0.01) hepatic lipid concentration compared with piglets fed CS milk replacer, regardless of prenatal choline status. Abbreviations: AHF, acid hydrolyzed fat; CD, choline deficient; CS, choline sufficient

Although there were no effects of perinatal choline status on liver weight as a percent of body weight, livers of piglets that received the postnatal CD treatment had a higher (*P* < 0.01) lipid concentration compared with piglets receiving the postnatal CS treatment, regardless of prenatal choline status (Fig 3).

### Metabolomics

Metabolomics analysis detected a total of 276 metabolites, of which 98 were significantly affected by the main effects of prenatal or postnatal treatment, or the interaction of prenatal and postnatal treatment (Table 5 and S2 Table). Unfortunately, treatment groups did not separate into distinct clusters using PCA plots. However, five main metabolic pathways were altered by perinatal choline status: one-carbon metabolism, membrane lipid metabolism, general lipid metabolism, glucose metabolism, and free fatty acid and dicarboxylate metabolism.

**Table 5:** Effects of perinatal choline status on metabolomic profiles of 4-wk-old piglets^1^.

		Treatment (Prenatal/Postnatal)					*P*-Value^2^		
Metabolite	Pathway	CS/CS	CS/CD	CD/CS	CD/CD	SEM	Pre	Post	Pre x Post
N-acetylmethionine	Amino Acid	1.00	0.94	0.74	0.86	0.08	0.03	0.69	0.27
Glutamine	Amino Acid	1.00	1.13	1.15	1.20	0.05	0.05	0.12	0.41
Glycine	Amino Acid	1.00	1.11	1.15	1.00	0.09	0.84	0.86	0.19
Histidine	Amino Acid	1.00	0.97	0.84	0.83	0.05	0.01	0.65	0.91
Lysine	Amino Acid	1.00	0.87	1.00	0.83	0.05	0.73	0.01	0.77
Phenylalanine	Amino Acid	1.00	0.96	0.86	0.79	0.06	0.02	0.35	0.82
Tryptophan	Amino Acid	1.00	1.01	0.88	0.93	0.04	0.01	0.46	0.70
Tyrosine	Amino Acid	1.00	1.08	0.70	0.85	0.07	<0.01	0.09	0.59
3-phosphoglycerate	Carbohydrate	1.00^a^	1.29^ab^	1.41^b^	1.05^a^	0.11	0.44	0.75	0.01
Glucose	Carbohydrate	1.00	0.85	0.89	0.86	0.05	0.35	0.10	0.29
Lactate	Carbohydrate	1.00	0.76	0.81	0.86	0.11	0.69	0.38	0.20
Phosphoenolpyruvate	Carbohydrate	1.00^a^	1.46^ab^	1.91^b^	1.07^a^	0.27	0.33	0.49	0.02
Pyruvate	Carbohydrate	1.00	1.08	1.29	1.11	0.14	0.26	0.70	0.36
1-linoleoylglycerophosphocholine	Lipid	1.00^b^	0.72^a^	0.57^a^	0.60^a^	0.06	<0.01	0.05	0.02
1-myristoylglycerophosphocholine	Lipid	1.00^b^	0.68^a^	0.55^a^	0.73^a^	0.09	0.03	0.46	0.01
1-palmitoleoylglycerophosphocholine	Lipid	1.00	0.64	0.59	0.56	0.07	<0.01	0.01	0.03
3-dehydrocarnitine	Lipid	1.00^ab^	1.49^c^	0.98^a^	1.18^b^	0.06	0.02	<0.01	0.04
3-hydroxybutyrate	Lipid	1.00	0.94	0.80	0.93	0.08	0.22	0.71	0.27
Acetylcarnitine	Lipid	1.00^a^	1.85^c^	0.88^a^	1.36^b^	0.08	<0.01	<0.01	0.03
Arachidonic Acid (20:4n6)	Lipid	1.00	1.50	1.07	1.22	0.13	0.42	0.02	0.20
Carnitine	Lipid	1.00	3.05	1.16	2.69	0.14	0.46	<0.01	0.06
Deoxycarnitine	Lipid	1.00^a^	1.25^b^	1.21^b^	0.98^a^	0.05	0.61	0.85	<0.01
Dimethylglycine	Lipid	1.00	0.10	0.87	0.17	0.06	0.63	<0.01	0.11
Docosahexaenoic Acid (22:6n3)	Lipid	1.00	1.71	1.05	1.25	0.16	0.21	0.01	0.13
Dodecanedioate	Lipid	1.00	1.23	1.01	1.20	0.10	0.92	0.05	0.85
Glycerol-3-phosphate	Lipid	1.00	0.16	0.89	0.33	0.33	0.92	0.04	0.67
Sarcosine	Lipid	1.00	0.66	1.02	0.58	0.11	0.76	<0.01	0.65
Adenosine 5’-monophosphate	Nucleotide	1.00^a^	2.42^ab^	2.78^b^	0.75^a^	0.57	0.92	0.60	0.01
Pantothenate	Vitamin	1.00^ab^	1.05^b^	1.06^b^	0.86^a^	0.05	0.21	0.18	0.03

^abc^Means within a row and without a common superscript differ (*P* < 0.05).

^1^Values are means of 8 replicate pigs exposed to prenatal and postnatal choline treatments (e.g., CS/CS as the control group) with blood collected from piglets at 27–30 d of age. Data presented as fold-change relative CS/CS treatment group. CD, choline deficient; CS, choline sufficient.

^2^Pre, main effect of prenatal choline status; Post, main effect of postnatal choline status; Pre x Post, interactive effect of prenatal and postnatal choline statuses.

#### One-carbon metabolism

There was a main effect of postnatal choline status on plasma choline, where postnatally CD piglets had lower (*P* < 0.01) choline than postnatally CS piglets (Fig 2B). Similarly, there was a main effect of postnatal choline status on plasma betaine where postnatally CD piglets had lower (*P* < 0.01) betaine than postnatally CS piglets (Fig 2C). Finally, there was a trend for prenatally CD piglets to have lower (*P* = 0.05) plasma methionine than prenatally CS piglets (Fig 2D).

#### Membrane lipid metabolism

There was a main effect of postnatal choline status on plasma glycerol-3-phosphate, where postnatally CD piglets had lower (*P* = 0.04) glycerol-3-phospate than postnatally CS piglets. Interactive effects (*P* < 0.05) between prenatal and postnatal choline status were observed for 1-linoleoylglycerophosphocholine and 1-palmitoleoylglycerophosphocholine, where piglets exposed to choline deficiency either prenatally or postnatally had lower concentrations than the CS/CS group (Table 5).

#### General lipid metabolism

There was a main effect of postnatal choline status on carnitine, where postnatally CD piglets had higher (*P* < 0.01) carnitine than postnatally CS piglets. Interactive effects (*P* < 0.05) between prenatal and postnatal choline status were observed for acetylcarnitine and 3-dehydrocarnitine, where CS/CS and CD/CS pigs were lowest and not different, CD/CD pigs were relatively higher, and CS/CD pigs were the highest among all groups (Table 5).

#### Glucose metabolism

There were no effects of prenatal or postnatal choline status on glucose per se, but other metabolites involved in glycolysis were altered by perinatal choline status. There was an interaction between prenatal and postnatal choline status on 3-phosphoglycerate and phosphoenolpyruvate (*P* < 0.05) where reciprocal prenatal and postnatal choline status (i.e., CS/CD and CD/CS) resulted in elevated concentrations of these metabolites (Table 5).

#### Free fatty acid and dicarboxylate metabolism

Main effects (*P* < 0.05) of postnatal choline status were observed for arachidonic acid, docosahexaenoic acid, and dodecanedionate, where postnatally CD piglets had higher concentrations than postnatally CS piglets (Table 5).

## Discussion

This study investigated the effects of perinatal choline deficiency on physiologic and metabolic patterns in the neonatal piglets. Specifically, we sought to determine whether prenatal or postnatal choline status was more important for overall health and metabolism of the neonatal piglet. Overall, we observed that postnatal choline status was most influential on general metabolism and health parameters at 4 weeks of age; however, there were many instances where both pre- and postnatal choline status were impactful. As such, two main findings were observed: 1) the neonatal piglet exhibits physiological effects of perinatal choline deficiency similar to humans and rodents and 2) prenatal choline status affects postnatal metabolic patterns.

### Piglets exhibit physiological effects of choline deficiency similar to humans and rodents

Regardless of prenatal treatment, piglets receiving CD milk replacer exhibited metabolomic profiles similar to adult humans exposed to choline deficiency [10]. Carnitine and its related metabolites were increased in plasma, and choline and its associated metabolites (e.g., betaine, dimethylglycine, and sarcosine) were decreased due to choline deficiency in both humans and piglets. It may be that these elevations in choline metabolites are due to increased use of choline in the PEMT pathway, as these are intermediates in the demethylation of choline [6].

It is well known that choline deficiency (often coupled with methionine deficiency) causes lipid accumulation in the liver of adult mice and humans [8, 9, 30]. We know that a choline deficient diet in early life can also result in hepatic steatosis in the piglet. Adequate choline is required for hepatic packaging of VLDL and excretion into general circulation. This lipid accumulation places stress on the liver, which can be characterized clinically by increased circulating liver enzymes. Additionally, the accumulation of hepatic lipid should be mirrored by decreased circulating cholesterol concentrations in postnatally deficient subjects. Indeed, in our study, piglets exposed to postnatal choline deficiency exhibited increased circulating liver enzymes (i.e., ALP and GGT), decreased plasma cholesterol concentrations, and increased circulating immunoglobulins, which suggests the young pig responded to choline deficiency in a similar fashion to rodents and humans.

Though many studies evaluate the effects of choline deficiency on the liver or brain, there is some indication that skeletal muscle may also be affected. Muscle damage has been demonstrated in a human choline deficiency study [10], where it was observed that a small portion of the study population developed muscle damage (elevated serum creatine phosphokinase), and the authors correlated changes in certain metabolites with this damage. In our study, perinatal choline deficiency appeared to result in muscle damage, as indicated by increased circulating creatine kinase (a marker of muscle damage) and AST in piglets exposed to differing choline status either prenatally and postnatally. Thus, piglets exhibited some of the same clinically-important changes as observed in human subjects, including increases in carnitine, glutamine, and glycine, and decreases in choline, sarcosine, and dimethylglycine. In a study conducted in a cell culture model [31], researchers observed that skeletal muscle cells grown in a choline deficient culture media exhibited lipid accumulation and altered choline uptake at both the cell surface and mitochondrial membranes. These results, coupled with our findings, suggest that increased circulating muscle-related enzymes may be due in part to lipotoxicity caused by fat accumulation in the muscle.

We speculate that skeletal muscle of piglets exposed to choline deficiency prenatally may be programmed to efficiently respond to choline deficiency postnatally, whereas pigs subjected to a choline sufficient uterine environment may exhibit abnormal muscle metabolism when exposed to postnatal choline deficiency. Thus, in terms of muscle and liver health biomarkers, and blood chemistry values, the piglet appears to be a sensitive model to study perinatal choline deficiency in humans. These observations warrant further investigation in the pig model, as skeletal muscle metabolism was not directly examined in the present study.

### Differential prenatal and postnatal choline exposure affect metabolomic profiles of piglets

In addition to alterations in choline metabolism caused by perinatal choline deficiency, changes in several other metabolic pathways were observed. Choline is an integral component of cell membranes due to the bipolar nature of choline-containing phospholipids, and exposure to choline deficiency during the perinatal period decreased the concentration of choline-containing lysolipids (similar to phospholipids, but only contain one fatty acid) in piglets. In addition to decreased lysolipids concentrations, CD/CS piglets also exhibited decreased circulating concentrations of ethanolamine-containing lysolipids compared with CS/CS piglets. Whereas lysolipid concentrations were decreased in general, glycerophosphorylcholine (a metabolite directly related to phospholipid metabolism) was not affected, indicating that decreased choline availability, rather than increased membrane lipid degradation, may be responsible for these changes. Taken together, these observations suggest that postnatal choline deficiency results in a specific reduction in choline-containing phospholipids, and prenatal choline deficiency results in universal membrane lipid reduction.

In addition to membrane lipid metabolism, general lipid metabolism was affected by perinatal choline deficiency. Specifically, postnatal deficiency resulted in pronounced accumulations of carnitine-related metabolites, which help to facilitate β-oxidation in the mitochondria. Increases in plasma long-chain fatty acid concentrations suggested a reduction in degradation and utilization of these fatty acids, which is consistent with carnitine-related metabolites remaining unconjugated and not facilitating transport into the mitochondria [32, 33]. These findings provide additional support for increased PEMT activity in postnatally CD piglets [34]. Dicarboxylic acids such as hexadecanedioate, produced by ω-oxidation in the endoplasmic reticulum and peroxisome and then degraded via β-oxidation, were also enriched in the plasma of postnatally CD piglets. Taken together, these data indicate that postnatal choline deficiency may result in impaired β-oxidation, which is consistent with previous observations [35] where those rats exposed to dietary choline deficiency had impaired mitochondrial function.

Lipid metabolism was impacted most strongly by choline deficiency; however, glucose utilization was also affected in the piglet. Previous studies have shown that choline deficiency can have impacts on glucose metabolism elicited as improved glucose tolerance and insulin sensitivity in *ob/ob* mice with hepatic lipidosis [36]. In the piglet, glycolytic intermediates such as 3-phosphoglycerate and phosphoenolpyruvate were differentially affected by choline deficiency. Specifically, CS/CD and CD/CS groups were associated with elevations in circulating glycolytic intermediates when compared with the CS/CS group, whereas within the prenatal deficiency groups, trending reductions in glycolytic intermediates were observed. Future studies in piglets would be valuable to specifically explore the impact of choline deficiency on glucose handling in models with digestion and metabolism more similar to humans.

Though lipid and glucose metabolism appear to be differentially altered by perinatal choline deficiency in the pig, amino acid metabolism appears to be primarily influenced by prenatal choline status. We observed a general decrease in circulating amino acid concentrations in prenatally deficient piglets, which may indicate an up regulation of amino acid metabolism to increase clearance from the blood. Because both diets were balanced for AA content and pigs had equal growth over the course of the study, it should be noted that these changes are not due to dietary differences in protein quality or availability. Especially interesting are the changes in tryptophan and tyrosine concentrations, which are precursors for the neurotransmitters serotonin and dopamine, respectively. Overall, concentrations of these amino acids, along with phenylalanine, the precursor for *de novo* tyrosine synthesis, were decreased, which may subsequently influence cognitive function. As such, decreased attention span has been demonstrated in humans with acutely-depleted tyrosine and catecholamine concentrations without accompanying learning and memory deficits [37], but differences may exist where depletion is caused by an underlying nutrient deficiency.

Our findings that perinatal choline status influence brain growth in the piglet provide a launching point for further studies, especially as this is one of few studies to report the influence of perinatal choline status on the entire brain. Currently, our laboratory uses methods to study *in vivo* neurodevelopment and cognition in the piglet [15, 17, 28, 29]. Use of the piglet model may allow these effects to be more accurately quantified through the use of highly-controlled dietary and neurodevelopmental studies.

### Conclusion

In conclusion, the piglet appears to be an appropriate translational animal model to bridge the gap between rodent and human research on the impact of prenatal and postnatal choline status. Not only are piglets very similar to humans in terms of metabolism and neurodevelopment, but here we show that they are also very similar to both humans and rodents regarding the effects of perinatal choline deficiency, as characterized by changes in liver health markers and metabolomic profiles. Additionally, this study provides evidence that the piglet may be a sensitive model to examine the effect of perinatal choline status on neurodevelopment. Future studies are warranted to elucidate the effects of perinatal choline status on learning and memory in this animal model that is so comparable to human development.

### Strengths and Limitations

The present study evaluated the impacts of pre- and postnatal choline status on physiology and metabolism in the neonatal piglet. As such, an important strength of this study was the ability to evaluate the impact of intervention timing on piglet metabolism. Most studies evaluate only the effect of maternal choline intake on the offspring. Additionally, this study in keeping with the recent call by the NIH to include both male and female subjects. Although metabolomics analysis is a powerful research tool, it is by its nature difficult to interpret. Because many metabolites were detected simultaneously, the possibility for false discovery does exist.

## Supporting Information

S1 Table Effects of perinatal choline status on clinical hematology profiles of 4-wk-old piglets^1^. ^1^Values are means of pigs exposed to prenatal and postnatal choline treatments (e.g., CS/CS as the control group) with blood collected from piglets at 27–30 d of age. Differences in treatment replications were due to clotting of blood samples. CD, choline deficient; CS, choline sufficient; Hb, hemoglobin; MCH, mean corpuscular hemoglobin; MCHC, mean corpuscular hemoglobin concentration; MCV; mean corpuscular volume; WBC, white blood cells. ^2^Pre, main effect of prenatal choline status; Post, main effect of postnatal choline status; Pre x Post, interactive effect of prenatal and postnatal choline statuses.
(PDF)

S2 Table Effects of perinatal choline status on metabolomic profiles of 4-wk-old piglets^1^. ^abc^Means within a row and without a common superscript differ (*P* < 0.05). ^1^Values are means of 8 replicate pigs exposed to prenatal and postnatal choline treatments (e.g., CS/CS as the control group) with blood collected from piglets at 27–30 d of age. Data presented as fold-change relative to CS/CS treatment group. CD, choline deficient; CS, choline sufficient. ^2^Pre, main effect of prenatal choline status; Post, main effect of postnatal choline status; Pre x Post, interactive effect of prenatal and postnatal choline statuses.
(PDF)

## References

[1] Zeisel SH, Blusztajn JK. Choline and human nutrition. Annu Rev Nutr. 1994;14:269–96. 7946521 10.1146/annurev.nu.14.070194.001413

[2] Institute of Medicine aNAoSU, editor. Dietary reference intakes for folate, thiamin, riboflavin, niacin, vitamin B12, pantothenic acid, biotin, and choline Washington DC: Natl Acad Pres; 1998. 23193625

[3] Jensen HH, Batres-Marquez SP, Carriquiry A, Schalinske KL. Choline in the diets of the US population: NHANES, 2003–2004. FASEB J. 2007;21(lb219).

[4] Boeke CE, Gillman MW, Hughes MD, Rifas-Shiman SL, Villamor E, Oken E. Choline intake during pregnancy and child cognition at age 7 years. Am J Epidemiol. 2013;177:1338–47. 10.1093/aje/kws395 23425631 PMC3676149

[5] Cheatham CL, Goldman CL, Fischer LM, da Costa KA, Reznick JS, Zeisel SH. Phosphatidylcholine supplementation in pregnant women consuming moderate-choline diets does not enhance infant cognitive function: a randomized, double-blind, placebo-controlled trial. Am J Clin Nutr. 2012;96:1465–72. 10.3945/ajcn.112.037184 23134891 PMC3497930

[6] Yan J, Jiang X, West AA, Perry CA, Malysheva OV, Devapatla S, et al Maternal choline intake modulates maternal and fetal biomarkers of choline metabolism in humans. Am J Clin Nutr. 2012;95:1060–71. 10.3945/ajcn.111.022772 22418088

[7] Yan J, Jiang X, West AA, Perry CA, Malysheva OV, Brenna JT, et al Pregnancy alters choline dynamics: results of a randomized trial using stable isotope methodology in pregnant and nonpregnant women. Am J Clin Nutr. 2013;98:1459–67. 10.3945/ajcn.113.066092 24132975 PMC6410899

[8] Zeisel SH, Blusztajn JK. Choline and human nutrition. Annu Rev Nutr. 1994;14:269–96. 7946521 10.1146/annurev.nu.14.070194.001413

[9] Zeisel SH, da Costa KA, Franklin PD, Alexander EA, Lamont JT, Sheard NF, et al Choline, an essential nutrient for humans. FASEB J. 1991;5:2093–8. 2010061

[10] Sha W, da Costa KA, Fischer LM, Milburn MV, Lawton KA, Berger A, et al Metabolomic profiling can predict which humans will develop liver dysfunction when deprived if dietary choline. FASEB J. 2010;24:2962–75. 10.1096/fj.09-154054 20371621 PMC2909293

[11] Pond WGaXGL. Of pigs and people 2nd ed. Southern LaLL, editor. New York: CRC Press; 2001.

[12] Moughan PJ, Birtles MJ, Cranwell PD, Smith WC, Pedraza M. The piglet as a model for studying aspects of digestion and absorption in milk-fed human infants. World Rev Nutr Diet. 1992; 67:40–113. 1557912 10.1159/000419461

[13] Miller ER, Ullrey DE. The pig as a model for human nutrition. Annu Rev Nutr. 1987; 7:361–82. 3300739 10.1146/annurev.nu.07.070187.002045

[14] Dobbing J, Sands J. Comparative aspects of the brain growth spurt. Early Hum Dev. 1979;3:79–83. 118862 10.1016/0378-3782(79)90022-7

[15] Dilger RN, Johnson RW. Behavioral assessment of cognitive function using a translational neonatal piglet model. Brain Behav Immun. 2010;24:1156–65. 10.1016/j.bbi.2010.05.008 20685307

[16] Rytych JL, Elmore MR, Burton MD, Conrad MS, Donovan SM, Dilger RN, et al Early life iron deficiency impairs spatial cognition in neonatal piglets. J Nutr. 2012;142:2050–6. 10.3945/jn.112.165522 23014488

[17] Elmore MR, Dilger RN, Johnson RW. Place and direction learning in a spatial T-maze task by neonatal piglets. Anim Cogn. 2012 4 17. 10.1007/s10071-012-0495-9 PMC3646386 22526690

[18] Kornegay ET, Meacham TN. Evaluation of supplemental choline for reproducing sows housed in total confinement on concrete or in dirt lots. J Anim Sci. 1973;37:506–9. 4795912 10.2527/jas1973.372506x

[19] Stockland WL, Blaylock LG. Choline requirement of pregnant sows and gilts under restricted feeding conditions. J Anim Sci. 1974;39:1113–16. 4475046 10.2527/jas1974.3961113x

[20] Pyapali GK, Turner DA, Willaism CL, Meck WH, Swartzwelder HS. Prenatal dietary choline supplementation decreases the threshold for induction of long-term potentiation in young adult rats. J Neurophysiol. 1998;79:1790–6. 9535948 10.1152/jn.1998.79.4.1790

[21] Meck WH, Williams CL. Simultaneous temporal processing is sensitive to prenatal choline availability in mature and aged rats. Neuroreport 1997;8:3045–51. 9331912 10.1097/00001756-199709290-00009

[22] Meck WH, Smith RA, Williams CL. Organizational changes in cholinergic activity and enhanced visuospatial memory as a function of choline administered prenatally or postnatally or both. Behav Neurosci. 1989;103:1234–41. 2610916 10.1037//0735-7044.103.6.1234

[23] Jakob S, Mosenthin R, Huesgen G, Kinkeldei J, Poweleit KJ. Diurnal pattern of choline concentrations in serum of pigs as influenced by dietary choline or lecithin intake. Z Ernahrungswiss. 1998;37:353–7. 9894684 10.1007/s003940050036

[24] NRC. 2012 Nutrient requirements of swine 11th rev. ed. Washington, DC: Natl Acad Press.

[25] Donovan SM, Mar MH, Zeisel SH. Choline and choline ester concentrations in porcine milk throughout lactation. J Nutr Biochem. 1997;8:603–7.

[26] Evans AM, DeHaven CD, Barret T, Mitchell M, Milgram E. Integrated, nontargeted ultrahigh performance liquid chromatography/electrospray ionization tandem mass spectroscopy platform for the identification and relative quantification of the small-molecule complement of biological systems. Anal Chem. 2009;81:6656–67. 10.1021/ac901536h 19624122

[27] Ohta T, Masutomi N, Tsutsui N, Sakairi T, Mitchell M, Milburn MV, et al Untargeted metabolomic profiling as an evaluative tool of fenofibrate-induced toxicology in Fischer 344 male rats. Toxicol Pathol. 2009;37:521–35. 10.1177/0192623309336152 19458390

[28] Conrad MS, Dilger RN, Johnson RW. Brain growth of the domestic pig (Sus scrofa) from 2 to 24 weeks of age: a longitudinal MRI study. Dev Neurosci. 2012; 34:291–8. 10.1159/000339311 22777003 PMC3646377

[29] Conrad MS, Dilger RN, Nickolls A, Johnson RW. Magnetic resonance imaging of the neonatal piglet brain. Pediatr Res. 2012;71:179–84. 10.1038/pr.2011.21 22258129

[30] Itagaki H, Shimizu K, Morikawa S, Ogawa K, Ezaki T. Morphological and functional characterization of non-alcoholic fatty liver disease induced by a methionine-choline-deficient diet in C57BL/6 mice. Int J Clin Exp Pathol. 2013;6:2683–96. 24294355 PMC3843249

[31] Michel V, Singh RK, Bakovic M. The impact of choline availability on muscle lipid metabolism. Food Funct. 2011;2:53–62. 10.1039/c0fo00069h 21773586

[32] Carter AL, Frenkel R. The relationship of choline and carnitine in the choline-deficient rat. J Nutr. 1978;108:1748–54. 712418 10.1093/jn/108.11.1748

[33] Corredor C, Mansbach C, Bressler R. Carnitine depletion in the choline-deficient state. Biochim Biophys Acta.1967;144:366–74. 6064613 10.1016/0005-2760(67)90165-8

[34] da Costa KA, Sanders LM, Fischer LM, Zeisel SH. Docosahexaenoic acid in plasma phosphatidylcholine may be a potential marker for in vivo phosphatidylethanolamine N-methyltransferase activity in humans. Am J Clin Nutr. 2011;93:968–74. 10.3945/ajcn.110.011064 21411618 PMC3076652

[35] Pacelli C, Coluccia A, Grattagliano I, Cocco T, Petrosillo G, Paradies G, et al Dietary choline deprivation impairs rat brain mitochondrial function and behavioral phenotype. J Nutr. 2010;140:1072–9. 10.3945/jn.109.116673 20357080

[36] Wu G, Zhang L, Li T, Lopaschuk G, Vance DE, Jacobs RL. Choline deficiency attenuates body weight gain and improves glucose tolerance in *ob/ob* mice. J Obes. 2012;2012:319172 10.1155/2012/319172 22778916 PMC3385711

[37] Matrenza C, Hughes JM, Kemp AH, Wesnes KA, Harrison BJ, Nathan PJ. Simultaneous depletion of serotonin and catecholamines impairs sustained attention in healthy female subjects without affecting learning and memory. J Psychopharmacol. 2004;18:21–31. 15107181 10.1177/0269881104040215

