# Supplementary material for: Moderate Perinatal Choline Deficiency Elicits Altered Physiology and Metabolomic Profiles in the Piglet
Source: PLoS One. 2015 Jul 21;10(7):e0133500. doi: 10.1371/journal.pone.0133500 (PMC4510435; doi:10.1371/journal.pone.0133500)
Supplement: S1 Table — 1Values are means of pigs exposed to prenatal and postnatal choline treatments (e.g., CS/CS as the control group) with blood collected from piglets at 27–30 d of age. Differences in treatment replications were due to clotting of blood samples. CD, choline deficient; CS, choline sufficient; Hb, hemoglobin; MCH, mean corpuscular hemoglobin; MCHC, mean corpuscular hemoglobin concentration; MCV; mean corpuscular volume; WBC, white blood cells. 2Pre, main effect of prenatal choline status; Post, main effect of postnatal choline status; Pre x Post, interactive effect of prenatal and postnatal choline statuses. (PDF) [file pone.0133500.s001.pdf]

S1 Table Effects of perinatal choline status on clinical hematology profiles of 4-wk-old piglets[1]

Variable	Treatment (Prenatal/Postnatal)				SEM	*P*-value[2]		
	CS/CS	CS/CD	CD/CS	CD/CD		Pre	Post	Pre x Post
n	8	8	5	6				
RBC, 10^{12}/L	5.2	5.4	5.4	5.4	0.2	0.56	0.29	0.60
Hb, mmol/L	5.54	5.79	5.70	5.78	0.17	0.61	0.25	0.55
Hematocrit, %	29	30	29	29	0.9	0.93	0.32	0.82
MCV, fL	55	54	54	54	0.9	0.33	0.85	0.53
MCH, pg	17	17	17	17	0.3	0.85	0.78	0.97
MCHC, mmol/L	312	316	318	317	20	0.05	0.40	0.09
WBC, 10^9/L	4.6	3.9	4.6	4.1	0.5	0.86	0.19	0.79
Neutrophils, %WBC	36.1	28.5	35.8	34.9	3.8	0.37	0.20	0.31
Lymphocytes, %WBC	59.8	69.5	62.0	62.6	4.2	0.52	0.16	0.22
Monocytes, %WBC	3.4	1.5	2.2	2.6	1.2	0.95	0.48	0.30
Platelets, $10^{9/}$L	276	255	325	324	43	0.14	0.78	0.79

[1]Values are means of pigs exposed to prenatal and postnatal choline treatments (e.g., CS/CS as the control group) with blood collected from piglets at 27-30 d of age. Differences in treatment replications were due to clotting of blood samples. CD, choline deficient; CS, choline sufficient; Hb, hemoglobin; MCH, mean corpuscular hemoglobin; MCHC, mean corpuscular hemoglobin concentration; MCV; mean corpuscular volume; WBC, white blood cells.

[2]Pre, main effect of prenatal choline status; Post, main effect of postnatal choline status; Pre x Post, interactive effect of prenatal and postnatal choline statuses.
