# Supplementary material for: Moderate Perinatal Choline Deficiency Elicits Altered Physiology and Metabolomic Profiles in the Piglet
Source: PLoS One. 2015 Jul 21;10(7):e0133500. doi: 10.1371/journal.pone.0133500 (PMC4510435; doi:10.1371/journal.pone.0133500)
Supplement: S2 Table — abcMeans within a row and without a common superscript differ (P < 0.05). 1Values are means of 8 replicate pigs exposed to prenatal and postnatal choline treatments (e.g., CS/CS as the control group) with blood collected from piglets at 27–30 d of age. Data presented as fold-change relative to CS/CS treatment group. CD, choline deficient; CS, choline sufficient. 2Pre, main effect of prenatal choline status; Post, main effect of postnatal choline status; Pre x Post, interactive effect of prenatal and postnatal choline statuses. (PDF) [file pone.0133500.s002.pdf]

S2 Table Effects of perinatal choline status on metabolomic profiles of 4-wk-old piglets[1]

Metabolite	Pathway	Treatment (Prenatal/Postnatal)				SEM	*P*-Value[2]		
		CS/CS	CS/CD	CD/CS	CD/CD		Pre	Post	Pre x Post
3-(4-hydroxyphenyl)lactate	Amino Acid	1.00	0.89	0.55	0.64	0.09	**<0.01**	0.90	0.27
3-hydroxy-2-ethylpropionate	Amino Acid	1.00	0.98	0.94	0.87	0.10	0.42	0.69	0.80
3-hydroxyisobutyrate	Amino Acid	1.00	0.94	0.80	0.93	0.08	0.22	0.71	0.27
3-indoxyl sulfate	Amino Acid	1.00	1.07	0.94	1.02	0.19	0.70	0.57	0.97
3-methoxytyrosine	Amino Acid	1.00	1.16	1.15	0.99	0.09	0.93	0.99	0.10
3-methyl-2-oxobutyrate	Amino Acid	1.00	1.12	1.12	1.10	0.12	0.72	0.68	0.58
3-methyl-2-oxovalerate	Amino Acid	1.00	1.03	1.03	0.94	0.08	0.71	0.75	0.47
4-hydroxyglutamate	Amino Acid	1.00	0.95	1.01	1.05	0.11	0.63	0.94	0.69
4-methyl-2-oxopentanoate	Amino Acid	1.00	1.13	1.25	1.17	0.10	0.16	0.78	0.32
5-oxoproline	Amino Acid	1.00	1.03	1.16	1.06	0.05	0.06	0.48	0.24
Alanine	Amino Acid	1.00	0.88	1.19	1.02	0.09	0.09	0.14	0.76
Alpha-hydroxyisovalerate	Amino Acid	1.00	0.76	0.63	0.96	0.14	0.52	0.74	0.05
Arginine	Amino Acid	1.00	0.92	0.89	0.88	0.07	0.30	0.52	0.66
Asparagine	Amino Acid	1.00	0.93	0.88	0.68	0.08	**0.03**	0.11	0.47
Aspartate	Amino Acid	1.00	0.86	0.80	0.72	0.22	0.45	0.60	0.89
Beta-alanine	Amino Acid	1.00	0.81	1.24	0.78	0.14	0.45	**0.03**	0.33
Beta-hydroxypyruvate	Amino Acid	1.00	0.75	0.85	0.82	0.08	0.65	0.10	0.19
Betaine	Amino Acid	1.00	0.23	0.94	0.20	0.04	0.26	**<0.01**	0.65
Carnosine	Amino Acid	1.00[a]	1.17[b]	1.12[ab]	0.99[a]	0.06	0.62	0.71	**0.01**
C-glycosyltryptophan	Amino Acid	1.00	1.05	1.08	0.97	0.04	0.97	0.52	0.04
Citrulline	Amino Acid	1.00	1.01	0.71	0.82	0.08	**<0.01**	0.44	0.53
Cysteine	Amino Acid	1.00	1.03	0.88	1.04	0.09	0.54	0.31	0.50
Gamma-glutamyl isoleucine	Amino Acid	1.00	0.87	0.86	0.78	0.06	0.09	0.10	0.68
Gamma-glutamyl leucine	Amino Acid	1.00	0.95	0.83	0.81	0.12	0.21	0.76	0.90
Gamma-glutamyl methionine	Amino Acid	1.00	0.91	1.32	0.85	0.12	0.27	**0.03**	0.13
Gamma-glutamyl phenylalanine	Amino Acid	1.00	0.86	0.80	0.71	0.07	**0.02**	0.11	0.75
Gamma-glutamyl tyrosine	Amino Acid	1.00	0.99	0.70	0.79	0.07	**<0.01**	0.59	0.47
Gamma-glutamyl valine	Amino Acid	1.00	0.80	0.84	0.80	0.09	0.38	0.18	0.36
Glutamate	Amino Acid	1.00	0.67	0.64	0.80	0.14	0.40	0.53	0.08
Glutamine	Amino Acid	1.00	1.13	1.15	1.20	0.05	**0.05**	0.12	0.41
Glycine	Amino Acid	1.00	1.11	1.15	1.00	0.09	0.84	0.86	0.19
Glycyclglycine	Amino Acid	1.00	1.19	1.12	1.05	0.12	0.92	0.60	0.27
Histidine	Amino Acid	1.00	0.97	0.84	0.83	0.05	**0.01**	0.65	0.91
Indolelactate	Amino Acid	1.00	0.90	0.73	0.62	0.11	**0.02**	0.35	0.95
Indolepropionate	Amino Acid	1.00	0.74	0.70	0.44	0.20	0.14	0.19	1.00
Isoleucine	Amino Acid	1.00	0.93	0.89	0.91	0.04	0.09	0.53	0.30

Isoleucylglycine	Amino Acid	1.00	1.07	1.01	0.99	0.07	0.62	0.72	0.56
Isovalerylglycine	Amino Acid	1.00	1.32	0.76	1.21	0.17	0.31	**0.03**	0.72
Kynurenine	Amino Acid	1.00	1.06	0.95	1.21	0.11	0.65	0.16	0.38
Leucine	Amino Acid	1.00	0.90	0.92	0.92	0.08	0.72	0.59	0.56
Levulinate (4-oxovalerate)	Amino Acid	1.00	1.24	1.07	1.03	0.09	0.43	0.27	0.11
Lysine	Amino Acid	1.00	0.87	1.00	0.83	0.05	0.73	**0.01**	0.77
Methionine	Amino Acid	1.00	1.04	0.89	0.97	0.04	**0.05**	0.18	0.68
N6-acetyllysine	Amino Acid	1.00	0.96	0.91	0.87	0.04	**0.03**	0.28	0.95
N-acetylalanine	Amino Acid	1.00	1.01	0.84	0.86	0.05	**<0.01**	0.73	0.95
N-acetylglycine	Amino Acid	1.00	1.65	1.12	1.63	0.19	0.79	**0.01**	0.72
N-acetylmethionine	Amino Acid	1.00	0.94	0.74	0.86	0.08	**0.03**	0.69	0.27
N-acetylornithine	Amino Acid	1.00	1.10	1.02	1.07	0.07	0.97	0.34	0.71
N-acetylserine	Amino Acid	1.00	1.38	1.13	1.18	0.10	0.72	**0.04**	0.09
N-acetylthreonine	Amino Acid	1.00	1.04	0.86	0.91	0.06	**0.03**	0.46	0.96
Ornithine	Amino Acid	1.00	0.81	0.79	0.78	0.13	0.38	0.46	0.52
p-cresol sulfate	Amino Acid	1.00	1.15	1.30	1.25	0.14	0.14	0.73	0.47
Phenol sulfate	Amino Acid	1.00	1.23	1.51	1.01	0.22	0.52	0.56	0.12
Phenylacetate	Amino Acid	1.00	1.23	1.24	1.19	0.13	0.44	0.52	0.28
Phenylacetylaspartate	Amino Acid	1.00	1.10	1.24	1.10	0.07	0.11	0.78	0.10
Phenylacetylglycine	Amino Acid	1.00	1.20	1.01	1.23	0.11	0.83	0.07	0.91
Phenylalanine	Amino Acid	1.00	0.96	0.86	0.79	0.06	**0.02**	0.35	0.82
Pipecolate	Amino Acid	1.00	1.01	1.00	0.87	0.05	0.15	0.23	0.16
Pro-hydroxy-pro	Amino Acid	1.00	1.13	1.34	1.29	0.08	**<0.01**	0.61	0.26
Proline	Amino Acid	1.00	0.94	0.99	0.92	0.03	0.62	0.06	0.73
Pyroglutamine	Amino Acid	1.00	1.19	0.84	1.05	0.12	0.21	0.10	0.98
Serine	Amino Acid	1.00	0.79	1.00	0.79	0.07	0.99	**<0.01**	0.99
S-methylcysteine	Amino Acid	1.00	1.03	1.03	1.09	0.06	0.52	0.49	0.87
Threonine	Amino Acid	1.00	1.06	0.87	0.96	0.10	0.27	0.47	0.90
Trans-4-hydroxyproline	Amino Acid	1.00	1.36	1.27	1.36	0.08	0.09	**0.01**	0.10
Tryptophan	Amino Acid	1.00	1.01	0.88	0.93	0.04	**0.01**	0.46	0.70
Tyrosine	Amino Acid	1.00	1.08	0.70	0.85	0.07	**<0.01**	0.09	0.59
Urea	Amino Acid	1.00	1.10	1.13	0.89	0.18	0.82	0.70	0.35
Valine	Amino Acid	1.00	0.92	0.90	0.91	0.05	0.29	0.50	0.36
Chenodeoxycholate	Bile Salt	1.00	2.90	0.61	0.53	0.89	0.13	0.32	0.27
Glycohyocholate	Bile Salt	1.00	0.70	0.45	6.50	2.73	0.34	0.30	0.26
Glycolate	Bile Salt	1.00	0.99	0.99	0.99	0.04	0.84	0.97	0.86
Hyocholate	Bile Salt	1.00	2.36	0.71	1.44	0.69	0.39	0.14	0.65
Hyodeoxycholate	Bile Salt	1.00	2.16	0.97	0.71	0.52	0.17	0.39	0.18
Taurochenodeoxycholate	Bile Salt	1.00	0.53	0.53	3.78	1.49	0.36	0.36	0.22
1,5-anhydroglucitol	Carbohydrate	1.00	1.13	1.16	1.24	0.07	0.06	0.13	0.69

3-phosphoglycerate	Carbohydrate	1.00^{a}	1.29^{ab}	1.41^{b}	1.05^{a}	0.11	0.44	0.75	**0.01**
Alpha-ketoglutarate	Carbohydrate	1.00	1.21	0.84	0.92	0.13	0.10	0.28	0.63
Citrate	Carbohydrate	1.00	0.94	0.82	0.83	0.08	0.09	0.79	0.64
Erythritol	Carbohydrate	1.00	0.97	0.88	0.99	0.11	0.63	0.72	0.55
Erythronate	Carbohydrate	1.00	1.02	0.95	0.97	0.04	0.27	0.69	0.97
Fructose	Carbohydrate	1.00	0.89	0.80	1.05	0.12	0.87	0.58	0.15
Fumarate	Carbohydrate	1.00	1.03	0.88	0.85	0.11	0.18	0.99	0.75
Galacitol	Carbohydrate	1.00	1.09	0.97	0.83	0.16	0.38	0.90	0.49
Glucose	Carbohydrate	1.00	0.85	0.89	0.86	0.05	0.35	0.10	0.29
Lactate	Carbohydrate	1.00	0.76	0.81	0.86	0.11	0.69	0.38	0.20
Lactose	Carbohydrate	1.00	0.98	0.97	0.72	0.28	0.60	0.63	0.69
Malate	Carbohydrate	1.00	0.58	0.46	0.48	0.19	0.10	0.32	0.25
Mannitol	Carbohydrate	1.00	0.95	1.08	0.83	0.11	0.85	0.20	0.38
Mannose	Carbohydrate	1.00	0.79	0.97	0.90	0.07	0.57	0.06	0.35
Phosphoenolpyruvate	Carbohydrate	1.00^{a}	1.46^{ab}	1.91^{b}	1.07^{a}	0.27	0.33	0.49	**0.02**
Pyruvate	Carbohydrate	1.00	1.08	1.29	1.11	0.14	0.26	0.70	0.36
Sorbitol	Carbohydrate	1.00	0.89	1.12	0.83	0.21	0.87	0.33	0.67
Succinate	Carbohydrate	1.00	0.32	0.26	0.24	0.35	0.25	0.33	0.36
Creatine	Creatine	1.00	1.44	0.94	1.01	0.12	**0.05**	**0.04**	0.14
Creatinine	Creatine	1.00	1.02	0.87	0.99	0.04	0.07	0.12	0.24
Guanidinoacetate	Creatine	1.00	0.94	1.09	0.93	0.11	0.71	0.35	0.69
Biliverdin	Hemoglobin	1.00	2.20	1.55	2.92	0.53	0.24	**0.02**	0.87
Heme	Hemoglobin	1.00	1.23	1.24	1.18	0.22	0.68	0.71	0.49
Myo-inositol	Inositol	1.00^{ab}	1.08^{ab}	1.11^{b}	0.92^{a}	0.06	0.70	0.37	**0.04**
Pinitol	Inositol	1.00	1.54	0.61	1.26	0.18	0.08	**<0.01**	0.77
1,2-propanediol	Ketone	1.00	2.61	1.84	2.09	0.47	0.74	0.06	0.16
3-hydroxybutyrate	Ketone	1.00	0.73	0.76	0.72	0.15	0.43	0.33	0.45
10-heptadecenoate (17:1n7)	Lipid	1.00	1.91	0.99	1.45	0.30	0.44	**0.03**	0.47
10-nonadecenoate (19:1n9)	Lipid	1.00	1.62	1.11	1.67	0.28	0.77	**0.04**	0.93
1-arachidonoylglycerophosphocholine	Lipid	1.00	0.91	0.64	0.87	0.10	**0.05**	0.48	0.08
1-arachidonoylglycerophosphoethanolamine	Lipid	1.00	1.82	0.85	1.62	0.13	0.20	**<0.01**	0.86
1-arachidonoylglycerophosphoinositol	Lipid	1.00^{a}	1.50^{b}	1.15^{ab}	1.08^{a}	0.13	0.31	0.11	**0.04**
1-docosahexaenolylglycerophosphocholine	Lipid	1.00	0.90	0.75	0.96	0.08	0.07	0.99	0.25
1-docosapentaenoylglycerophosphocholine	Lipid	1.00	0.71	0.61	0.52	0.10	**0.01**	0.06	0.32
1-eicosadienoylglycerophosphocholine	Lipid	1.00	0.64	0.65	0.52	0.11	**0.03**	**0.03**	0.30
1-eicosatrienoylglycerophosphocholine	Lipid	1.00	0.90	0.63	0.72	0.11	**0.02**	0.94	0.39
1-heptadecanoylglycerophosphocholine	Lipid	1.00	0.64	0.44	0.41	0.15	**0.01**	0.20	0.28
1-linoleoylglycerol	Lipid	1.00^{b}	0.72^{a}	0.57^{a}	0.60^{a}	0.06	**<0.01**	**0.05**	**0.02**
1-linoleoylglycerophosphocholine	Lipid	1.00^{b}	0.72^{a}	0.57^{a}	0.60^{a}	0.06	**<0.01**	**0.05**	**0.02**
1-linoleoylglycerophosphoethanolamine	Lipid	1.00	1.32	0.66	0.92	0.11	**<0.01**	**0.01**	0.79

1-methylimidazoleaceate	Lipid	1.00	0.98	1.05	1.03	0.11	0.64	0.83	0.99
1-myristoylglycerophosphocholine	Lipid	1.00[b]	0.68[a]	0.55[a]	0.73[a]	0.09	**0.03**	0.46	**0.01**
1-oleoylglycerophosphocholine	Lipid	1.00	0.54	0.68	0.54	0.08	**0.05**	**<0.01**	**0.05**
1-oleoylglycerophosphoethanolamine	Lipid	1.00	1.32	0.70	0.88	0.12	**0.01**	**0.05**	0.58
1-palmitoleoylglycerophosphocholine	Lipid	1.00	0.64	0.59	0.56	0.07	**<0.01**	**0.01**	**0.03**
1-palmitoylglycerophosphocholine	Lipid	1.00	0.63	0.66	0.58	0.08	**0.03**	**0.01**	0.09
1-palmitoylglycerophosphoethanolamine	Lipid	1.00	1.01	0.73	0.87	0.09	0.03	0.41	0.47
1-palmitoylplasmenylethanolamine	Lipid	1.00	0.95	1.06	0.90	0.11	0.94	0.36	0.65
1-stearoylglycerophosphocholine	Lipid	1.00	0.81	0.34	0.66	0.14	**0.01**	0.63	0.08
1-stearoylglycerophosphoethanolamine	Lipid	1.00	1.06	0.91	1.11	0.08	0.79	0.12	0.42
1-stearoylglycerophosphoinositol	Lipid	1.00	1.49	0.96	1.37	0.13	0.54	**<0.01**	0.75
2,3-diphosphoglycerate	Lipid	1.00[ab]	1.19[ab]	1.88[b]	0.78[a]	0.30	0.45	0.15	**0.04**
2-aminoadipate	Lipid	1.00	0.61	0.56	0.61	0.12	0.07	0.14	0.07
2-aminobutyrate	Lipid	1.00	1.13	1.06	1.14	0.08	0.66	0.19	0.73
2-arachidonoylglycerophosphoethanolamine	Lipid	1.00	1.75	0.92	1.46	0.11	0.10	**<0.01**	0.36
2-ethylhexanoate	Lipid	1.00	0.83	0.91	0.70	0.08	0.18	**0.02**	0.78
2-hydroxybutyrate	Lipid	1.00	0.97	0.81	0.95	0.16	0.53	0.75	0.62
2-hydroxyglutarate	Lipid	1.00	0.87	0.60	0.72	0.11	**0.02**	0.97	0.25
2-hydroxyisobutyrate	Lipid	1.00	0.86	0.95	0.86	0.06	0.67	0.07	0.71
2-hydroxypalmitate	Lipid	1.00	1.50	1.28	1.34	0.16	0.70	0.08	0.17
2-linoleoylglycerophosphocholine	Lipid	1.00	0.66	0.57	0.50	0.08	**<0.01**	**0.01**	0.09
2-linoleoylglycerophosphoethanolamine	Lipid	1.00	1.29	0.69	0.90	0.11	**<0.01**	**0.03**	0.69
2-oleoylglycerophosphoethanolamine	Lipid	1.00	1.21	0.65	0.78	0.12	**<0.01**	0.16	0.77
2-palmitoylglycerophosphocholine	Lipid	1.00[b]	0.59[a]	0.57[a]	0.55[a]	0.08	**<0.01**	**0.01**	**0.01**
2-stearoylglycerophosphocholine	Lipid	1.00	0.65	0.55	0.55	0.15	0.07	0.25	0.25
3-dehydrocarnitine	Lipid	1.00[ab]	1.49[c]	0.98[a]	1.18[b]	0.06	**0.02**	**<0.01**	**0.04**
3-hydroxybutyrate	Lipid	1.00	0.94	0.80	0.93	0.08	0.22	0.71	0.27
3-hydroxypropanoate	Lipid	1.00	0.70	0.87	1.48	0.35	0.36	0.65	0.20
3-phosphoglycerate	Lipid	1.00[a]	1.29[ab]	1.41[b]	1.05[a]	0.11	0.44	0.75	**0.01**
7-alpha-hydroxy-3-oxo-4-cholestenoate	Lipid	1.00	1.92	1.26	2.52	0.81	0.60	0.19	0.84
7-beta-hydroxycholesterol	Lipid	1.00	0.70	1.14	0.87	0.12	0.24	**0.03**	0.92
Acetylcarnitine	Lipid	1.00[a]	1.85[c]	0.88[a]	1.36[b]	0.08	**<0.01**	**<0.01**	**0.03**
Adrenic Acid (22:4n6)	Lipid	1.00	1.22	1.25	1.14	0.12	0.48	0.65	0.17
Alpha-linoelnic Acid (18:3n3)	Lipid	1.00	2.27	0.88	1.20	0.36	0.11	**0.04**	0.20
Arachidonic Acid (20:4n6)	Lipid	1.00	1.50	1.07	1.22	0.13	0.42	**0.02**	0.20
Caprylate (10:0)	Lipid	1.00	1.13	1.05	0.96	0.10	0.59	0.86	0.29
Carnitine	Lipid	1.00	3.05	1.16	2.69	0.14	0.46	**<0.01**	0.06
Cholesterol	Lipid	1.00	0.93	1.00	0.94	0.05	0.85	0.21	0.91
Choline	Lipid	1.00	0.54	0.94	0.45	0.05	0.13	**<0.01**	0.72
Cis-vaccenate (18:1n7)	Lipid	1.00	1.13	0.96	1.16	0.12	0.94	0.18	0.78

Deoxycarnitine	Lipid	1.00[a]	1.25[b]	1.21[b]	0.98[a]	0.05	0.61	0.85	**<0.01**
Dimethylglycine	Lipid	1.00	0.10	0.87	0.17	0.06	0.63	**<0.01**	0.11
Docosadienoic Acid (22:2n6)	Lipid	1.00	1.63	1.34	1.73	0.19	0.25	**0.01**	0.54
Docosahexaenoic Acid (22:6n3)	Lipid	1.00	1.71	1.05	1.25	0.16	0.21	**0.01**	0.13
Docosapentanoic Acid (22:5n6)	Lipid	1.00	1.37	1.31	1.38	0.20	0.44	0.30	0.47
Dodecanedioate	Lipid	1.00	1.23	1.01	1.20	0.10	0.92	**0.05**	0.85
Eicosadienoic Acid (20:2n6)	Lipid	1.00	1.83	1.30	1.93	0.35	0.58	**0.05**	0.78
Eicosanoate (20:1n9/n11)	Lipid	1.00	2.24	1.31	2.42	0.49	0.62	**0.02**	0.89
Eicosapentaenoic Acid (22:5n3)	Lipid	1.00	1.35	1.22	1.22	0.16	0.78	0.27	0.27
Eicosatrienoic acid (20:3n3/n6)	Lipid	1.00	1.65	1.35	1.77	0.24	0.33	**0.03**	0.64
Glycerate	Lipid	1.00	1.05	1.06	0.94	0.08	0.80	0.67	0.33
Glycerol	Lipid	1.00	0.87	0.83	0.87	0.10	0.40	0.66	0.35
Glycerol-3-phosphate	Lipid	1.00	0.16	0.89	0.33	0.33	0.92	**0.04**	0.67
Glycerophosphocholine	Lipid	1.00	0.74	0.73	0.58	0.18	0.25	0.27	0.75
Heptanoate (7:0)	Lipid	1.00	1.10	1.15	1.08	0.09	0.46	0.83	0.37
Lathosterol	Lipid	1.00	0.85	0.74	0.93	0.12	0.49	0.88	0.18
Laurate (12:0)	Lipid	1.00	1.03	0.93	0.83	0.09	0.17	0.70	0.51
Linoleic Acid (18:2n6)	Lipid	1.00	1.99	0.89	1.23	0.32	0.19	**0.05**	0.32
Margarate (17:0)	Lipid	1.00	1.25	0.99	1.14	0.11	0.58	0.07	0.63
Methyl-stearate	Lipid	1.00	0.55	1.05	0.63	0.20	0.73	**0.03**	0.94
Myristate (14:0)	Lipid	1.00	1.44	0.91	0.99	0.15	0.08	0.08	0.24
Myristoleate (14:1n5)	Lipid	1.00	0.97	0.91	0.80	0.09	0.14	0.43	0.64
Nonadecanoate (19:0)	Lipid	1.00	1.34	1.18	1.23	0.12	0.79	0.13	0.25
Oleate (18:1n9)	Lipid	1.00	1.39	0.94	1.34	0.19	0.77	**0.05**	0.98
Palmitate (16:0)	Lipid	1.00	1.35	1.01	1.27	0.14	0.82	**0.04**	0.75
Palmitoleate (16:1n7)	Lipid	1.00	2.38	0.94	1.28	0.34	0.10	**0.02**	0.14
Palmitoyl sphingomyelin	Lipid	1.00	0.85	1.11	1.08	0.09	0.07	0.34	0.51
Pelargonate (9:0)	Lipid	1.00	1.08	1.07	0.98	0.09	0.88	0.94	0.35
Propionylcarnitine	Lipid	1.00	1.56	0.67	1.38	0.14	0.08	**<0.01**	0.61
Sarcosine	Lipid	1.00	0.66	1.02	0.58	0.11	0.76	**<0.01**	0.65
Stearate (18:0)	Lipid	1.00	1.35	1.04	1.34	0.12	0.90	**0.01**	0.83
Stearoyl sphingomyelin	Lipid	1.00	0.94	1.09	1.17	0.12	0.19	0.96	0.56
Succinylcarnitine	Lipid	1.00[a]	1.44[b]	1.24[ab]	1.23[ab]	0.09	0.87	**0.02**	**0.02**
Undecanoate (11:0)	Lipid	1.00	1.05	1.16	1.16	0.08	0.10	0.75	0.75
2’-deoxycytidine	Nucleotide	1.00	1.08	1.06	0.95	0.06	0.53	0.84	0.12
2’-deoxyuridine	Nucleotide	1.00	0.88	1.20	0.92	0.13	0.37	0.14	0.55
5,6-dihydrouracil	Nucleotide	1.00	1.02	1.34	1.05	0.11	0.10	0.24	0.17
Adenine	Nucleotide	1.00	0.83	0.94	0.75	0.11	0.51	0.10	0.91
Adenosine 5’-monophosphate	Nucleotide	1.00[a]	2.42[ab]	2.78[b]	0.75[a]	0.57	0.92	0.60	**0.01**
Allantoin	Nucleotide	1.00	0.99	1.06	1.05	0.08	0.45	0.92	1.00

Cytidine	Nucleotide	1.00	0.73	0.85	0.79	0.10	0.64	0.12	0.31
Dihydroorotate	Nucleotide	1.00	0.82	0.92	0.54	0.09	**0.04**	**<0.01**	0.26
Flavin-adenine dinucleotide (FAD)	Nucleotide	1.00	0.98	1.21	0.94	0.07	0.25	**0.05**	0.09
Guanine	Nucleotide	1.00	0.63	0.72	0.69	0.11	0.34	0.09	0.15
Guanosine	Nucleotide	1.00	0.67	0.66	0.54	0.22	0.29	0.32	0.64
Hypoxanthine	Nucleotide	1.00	0.83	0.81	0.75	0.07	0.07	0.13	0.47
Inosine	Nucleotide	1.00	1.18	1.03	0.79	0.17	0.29	0.88	0.23
Methylguanine7	Nucleotide	1.00	0.98	0.89	0.90	0.10	0.31	0.97	0.90
N1-methyladenosine	Nucleotide	1.00	1.10	0.93	1.06	0.05	0.32	**0.05**	0.82
N1-methylguanosine	Nucleotide	1.00	1.03	1.16	1.07	0.09	0.29	0.75	0.52
N2-methylguanosine	Nucleotide	1.00	1.07	0.99	0.90	0.05	0.07	0.91	0.12
N4-acetylcytidine	Nucleotide	1.00	1.29	0.98	1.06	0.10	0.23	0.08	0.30
N6-carbamoylthreonyladenosine	Nucleotide	1.00	0.98	0.93	1.02	0.05	0.78	0.51	0.30
Pseudouridine	Nucleotide	1.00	1.07	1.08	1.02	0.03	0.66	0.79	**0.05**
Ribitol	Nucleotide	1.00	0.63	0.56	0.58	0.13	0.08	0.20	0.15
Ribose	Nucleotide	1.00	0.98	1.11	0.88	0.14	0.98	0.39	0.46
Ribulose	Nucleotide	1.00	0.78	0.71	0.63	0.11	**0.05**	0.19	0.54
Threitol	Nucleotide	1.00	1.07	1.08	1.03	0.05	0.70	0.86	0.18
Thymidine	Nucleotide	1.00	0.97	1.10	0.99	0.10	0.55	0.48	0.65
Urate	Nucleotide	1.00	0.56	0.51	0.73	0.19	0.40	0.56	0.09
Uridine	Nucleotide	1.00	0.85	0.92	0.88	0.07	0.74	0.20	0.44
Xanthine	Nucleotide	1.00	0.59	0.53	0.64	0.22	0.36	0.50	0.25
Xylitol	Nucleotide	1.00	0.88	1.00	0.83	0.08	0.72	0.08	0.75
Xylonate	Nucleotide	1.00	0.90	0.92	0.86	0.07	0.41	0.22	0.75
Xylose	Nucleotide	1.00	0.85	1.04	0.81	0.07	0.93	**0.01**	0.56
4-hydroxyhippurate	Other	1.00[c]	0.81[b]	0.66[a]	0.67[a]	0.04	**<0.01**	**0.04**	**0.03**
Benzoate	Other	1.00	1.15	1.01	1.11	0.07	0.87	0.10	0.77
Benzyl alcohol	Other	1.00	7.50	3.29	6.11	2.06	0.83	**0.03**	0.38
Catechol sulfate	Other	1.00	1.01	0.68	0.99	0.13	0.18	0.21	0.25
Cinnamoylglycine	Other	1.00	1.09	0.88	1.77	0.32	0.38	0.13	0.22
EDTA	Other	1.00	1.11	1.01	1.37	0.15	0.36	0.13	0.39
Gulono-1,4-lactone	Other	1.00	1.03	1.01	1.18	0.13	0.56	0.47	0.61
Hippurate	Other	1.00[b]	0.57[ab]	0.35[a]	0.81[ab]	0.21	0.35	0.96	**0.04**
Homostachydrine	Other	1.00	0.61	1.07	0.58	0.04	0.74	**<0.01**	0.26
Iminodiacetate	Other	1.00	1.23	1.13	1.45	0.13	0.18	**0.04**	0.72
Inosine-5’-diphosphate	Other	1.00	1.05	0.92	0.92	0.09	0.24	0.74	0.80
Ketamine	Other	1.00	1.68	1.67	1.33	0.28	0.57	0.56	0.08
Oxalate (ethanedioate)	Other	1.00	1.00	0.82	0.87	0.12	0.21	0.81	0.83
Phosphate	Other	1.00	1.00	1.07	1.02	0.03	0.13	0.34	0.33
Pyrophosphate	Other	1.00	1.22	1.20	1.24	0.13	0.41	0.33	0.50

Threonate	Other	1.00	0.98	1.04	1.09	0.11	0.46	0.89	0.73
Alpha-tocopherol	Vitamin	1.00	0.55	0.76	0.74	0.12	0.85	0.06	0.08
Nicotinamide	Vitamin	1.00	1.11	0.95	1.07	0.10	0.69	0.26	0.93
Pantothenate	Vitamin	1.00[ab]	1.05[b]	1.06[b]	0.86[a]	0.05	0.21	0.18	**0.03**
Quinolinate	Vitamin	1.00	1.12	1.07	1.34	0.17	0.79	0.59	0.87

[abc]Means within a row and without a common superscript differ ($P < 0.05$).

[1]Values are means of 8 replicate pigs exposed to prenatal and postnatal choline treatments (e.g., CS/CS as the control group) with blood collected from piglets at 27-30 d of age. Data presented as fold-change relative to CS/CS treatment group. CD, choline deficient; CS, choline sufficient.

[2]Pre, main effect of prenatal choline status; Post, main effect of postnatal choline status; Pre x Post, interactive effect of prenatal and postnatal choline statuses.
